# The hidden burden of viral hepatitis: a comparative study of KAP and infections among rohingya refugees and host communities in Bangladesh

**DOI:** 10.1186/s12889-026-27880-6

**Published:** 2026-05-25

**Authors:** Shoeb Bin Islam, Md Fuad Al Fidah, Tanvir Ahmed Rasel, Sharika Nuzhat, Monira Sarmin, Mohammod Jobayer Chisti, Tahmeed Ahmed, M Munirul Islam

**Affiliations:** 1 Nutrition Research Division, icddr,b, Dhaka, 1212 Bangladesh; 2 Office of the Executive Director, icddr,b, Dhaka, 1212 Bangladesh

**Keywords:** HBV, HCV, FDMNs, KAP score, Host community

## Abstract

**Background:**

Bangladesh has an intermediate endemicity of hepatitis B virus (HBV), with prevalence exceeding the global average. In border areas like Teknaf, where more than a million Forcibly Displaced Myanmar Nationals (FDMNs), as well as the Local Community Bangladeshi Nationals (host community), may be at increased risk of HBV and hepatitis C virus (HCV). We aimed to explore the knowledge, attitudes, and practices (KAP) regarding HBV and HCV in these populations.

**Methods:**

This was a cross-sectional study among adults who reported to the Severe Acute Respiratory Infection (SARI) Hospital in Teknaf, Cox’s Bazar, between April 2021 and March 2022. A structured questionnaire was developed to assess the participants’ knowledge, attitudes, and practices on HBV or HCV. Simultaneously, blood specimens from the participants were analysed to explore their infection status. KAP scores were calculated by assigning one point for each correct response, and participants were classified as satisfactory or unsatisfactory based on a midpoint cut-off of the total possible score. A p-value of < 0.05 was considered significant.

**Results:**

A total of 654 participants were enrolled; 58 (8.86%) were FDMNs and 596 (91.14%) were host community. Overall, 23 (3.52%) tested positive for either HBV or HCV, including 11 (18.97%) FDMNs and 12 (2.01%) host community (*p* < 0.001). HBV positivity was comparable between FDMNs and host community (1.72% vs. 1.01%; *p* = 0.480). HCV positivity was higher among FDMNs than host community (17.24% vs. 1.01%; *p* < 0.001). Satisfactory knowledge was observed in 5.17% of FDMNs and 12.25% of host community; satisfactory attitude in 71.70% and 81.12%, respectively; and satisfactory practice in 37.93% and 50.00%. FDMNs had poorer awareness of HBV vaccination (0.00% vs. 9.73%) and vertical transmission (6.90% vs. 19.33%).

**Conclusion:**

KAP regarding HBV and HCV was low in both populations, particularly among FDMNs, who also had a substantially higher infection burden. Targeted health education, culturally appropriate awareness programmes, and improved access to screening and care may help reduce future viral hepatitis transmission in both communities.

**Supplementary Information:**

The online version contains supplementary material available at 10.1186/s12889-026-27880-6.

## Introduction

According to the World Health Organisation (WHO), an estimated 296 million people were living with hepatitis B (HBV) and 58 million with hepatitis C (HCV) in 2019, representing about 4.4% of the global population [1]. The organisation has set the goal of eliminating viral hepatitis as a public health threat by 2030 [1]. Hepatitis B (HBV) and hepatitis C (HCV) remain major causes of chronic liver disease in Bangladesh. In 2017, nearly one million Forcibly Displaced Myanmar Nationals fled Rakhine State and settled in temporary camps in Cox’s Bazar [2]. A recent study reported a high prevalence of HCV infection among FDMNs, indicating a substantial burden of viral hepatitis within this population [3].

HBV prevalence is categorised by the WHO as low (up to 2%), intermediate (2–8%), or high (> 8%) [4]. Bangladesh is a country where the prevalence of HBV infection is considered to be intermediate (4.0%), which is still higher than the global prevalence (3.5%) [5]. The estimated prevalence of HCV is 0.88% [6]. A survey of 641 FDMNs reported that 30.4% tested positive for HCV and 19.8% had an active infection [7]. In Cox’s Bazar, HCV was found in 8.0% of the pregnant FDMNs [2]. Differences in prevalence estimates across studies may reflect variations in study populations, sample sizes, and diagnostic methods.

Patients infected with HBV and HCV are often asymptomatic until they show signs of decompensation. HBV and HCV share a common transmission mode but differ in transmissibility [8]. The risk of exposure and disease transmission may be increased by certain behaviors, including having sex with multiple sexual partners and intravenous drug abuse, having tattoos or piercing ears, etc. [9]. Cross-border population movement and close interaction between FDMNs and host community may facilitate the transmission of infectious diseases between the two populations [10]. A KAP survey is a structured quantitative research method that gathers information through the administration of standardized, pre-formulated questionnaires [11]. KAP surveys can identify knowledge gaps and barriers and help inform targeted interventions.

## Methodology

### Study site

A 60-bed hospital was established by icddr, b in collaboration with UNICEF to provide healthcare to both host community and FDMNs in Teknaf, Cox’s Bazar. Healthcare providers from icddr, b cared for a variety of conditions, including diarrhoea, severe acute malnutrition, and severe respiratory tract infections. With an average of 100 to 150 outpatients and 12 to 15 inpatients per day, the facility offers both inpatient and outpatient services. Basic laboratory tests can be performed by the well-equipped and skilled staff of the Laboratory Service Department.

### Study population

Adult patients (≥ 18 years) or carers who visited the icddr, b Teknaf Hospital for treatment and gave written consent, regardless of national origin or sex, were included in this study. Participants were recruited from a SARI-designated hospital in Teknaf, which serves both FDMN and host community populations. This facility-based approach enabled the inclusion of individuals from both communities within the same setting. However, as participants were recruited using convenience sampling from a hospital setting, the findings may not be representative of the general population and selection bias cannot be excluded.

### Sample size

This study used continuous convenience sampling to collect data from the adult patients who reported to the hospital. The study period was April 1, 2021 to March 31, 2022. Finally, data from 654 participants were analysed which included data from 596 host community and 58 FDMNs. The relatively lower representation of FDMNs reflects reduced attendance by FDMNs at this facility during the study period, potentially due to differences in healthcare access and utilisation. The smaller number of FDMNs may also have limited statistical precision and reduced the ability to detect smaller between-group differences. Participants were enrolled regardless of hepatitis status and included individuals attending for various clinical indications.

### Data collection and measurement

Before data collection, informed written consent was obtained from the participants. The attending physician then completed the case-record forms (in Bangla and Rohingya languages). Data were collected in a separate room within the hospital to ensure privacy. For information regarding sexual behavior, a study physician of the same sex conducted the interview. The medical examination was conducted by the study physician. The questionnaire was administered by trained field workers familiar with the local language and cultural context. Field workers received training on interview procedures and standardised administration. The questionnaire was pilot tested in Bangla and English among 30 volunteers prior to the main study to assess comprehension and contextual appropriateness. Formal analysis of pilot data was not performed, and these data were not included in the final analysis. During data collection, the questionnaire was explained verbally in a language understood by participants, including Bangla or Rohingya as appropriate, and clarifications were provided as needed to ensure comprehension, particularly among Rohingya participants. Although formal back-translation was not conducted, efforts were made to ensure linguistic and contextual appropriateness.The tool was administered by trained bilingual research assistants to ensure conceptual equivalence in the Rohingya dialect.

### Laboratory procedures

Approximately 3 ml of venous blood was collected from each participant. Serum or plasma samples were tested within 8 h using rapid chromatographic immunoassays. Hepatitis B surface antigen (HBsAg) was detected using the SD BIOLINE HBsAg rapid test (Standard Diagnostics Inc., Republic of Korea; sensitivity 99.5%, specificity 100%). Anti-HCV antibodies were detected using the ACTIVE HCV rapid test cassette (Hangzhou AllTest Biotech Co., Ltd., China; sensitivity 98.8%, specificity 99.1%). Tests were performed according to manufacturer instructions, and results were interpreted based on the presence or absence of control and test lines. These rapid immunoassays were used as screening tools, and confirmatory ELISA or PCR testing was not performed and were chosen for feasibility in a resource-limited field setting.

### Knowledge, attitude, and practice toward HBV and HCV

KAP surveys assess what people know, their beliefs, and what they do regarding a specific health issue or behaviour. These surveys collect data in three main areas: practices, attitudes (beliefs, values, and perceptions), and knowledge (awareness and understanding) [12]. A total of 49 items (17 for knowledge, 25 for attitude, and 7 for practice) were used to gauge participants’ KAP regarding HBV and HCV. After a thorough review of the literature, this questionnaire was developed [13–15]. The questionnaire was then pretested on 30 volunteers. Data from the pre-testing were not included in the final analysis. The internal validity was examined using Cronbach’s alpha = 0.84, indicating good internal consistency [16]. For external validity, experts at icddr, b in Dhaka, Bangladesh, were consulted.

### Knowledge about HBV and HCV

Seventeen questions about aetiology, transmission, and vaccination were used to assess knowledge. Each correct response received a score of 1. Incorrect answers were scored as 0. The range of the total knowledge score was 0–17. Following a previously described midpoint approach, the midpoint of the possible range was selected as a cut-off [13]. All items were assigned equal weight, consistent with previously published KAP studies using midpoint-based categorisation. As such, knowledge scores less than 8 were considered ‘unsatisfactory’ and scores ranging 8–17 as ‘satisfactory’.

### Attitudes about HBV and HCV

To evaluate the attitude score, 25 questions were used, scoring 1 for a positive attitude and 0 for a negative attitude. The final score ranged from 0 to 25. Participants with an attitude score greater than 12 (the midpoint of the possible range) were considered “satisfactory”, while all others were considered “unsatisfactory”. All attitude items were equally weighted.

### Preventive practices about HBV and HCV

To assess the participants’ preventive practices regarding HBV and HCV, we used 7 questions with a range of 0 to 7. Good practice was scored as 1, and poor practice as 0. Participants with a practice score of > 3 were considered “satisfactory”. All practice items were equally weighted for score calculation.

### Evaluation of viral markers

All the participants in the study had blood tests done. The sample was taken to the lab immediately, where lab personnel performed immunoassay tests for HBV and HCV. The participant ID number was used to record the results into the CRF. The study nurse informed each person who took the test about the results. If the results were positive, the patient was sent to a doctor for more counselling and advice on how to handle the situation. People who had HBV or HCV were advised on how to prevent infection and referred to the appropriate medical facilities.

### Data analysis

Data were analysed with STATA 15 (StataCorp, College Station, Texas, USA). Statistical analysis encompassed descriptive methods, incorporating percentages of characteristics, responses, and infection status. Responses to KAP items were categorised as ‘yes’, ‘no’, ‘cannot explain properly’, or ‘refused to answer’. For analysis, we included only cases with positive or negative responses. Mean and standard deviation (SD) were used to summarise normally distributed quantitative data, while median and interquartile range (IQR) values were used to summarise non-normally distributed data. Where applicable, associations were examined using chi-square, Fisher’s exact, t-test or the Mann-Whitney U test. Simple logistic regression analysis was conducted to explore the association of type of residency with infection types. The type of residency was coded as a binary variable, with host community as the reference category and FDMNs as the comparison group. The odds ratios represent the likelihood of being FDMN relative to host community given the exposure. Unadjusted Odds Ratios (uOR) and their corresponding 95% CI were reported. A p-value < 0.05 was considered statistically significant. Due to the limited number of outcome events, multiple logistic regression was not performed to avoid overfitting and unstable estimates.

## Results

A total of 58 FDMNs and 596 host community were included in the study. Among these participants, 11 (18.97%) FDMNs and 12 (2.01%) host community tested positive for either HBV or HCV infections, while no participants had simultaneous HBV and HCV coinfection. The prevalence of HBV or HCV positivity was significantly higher among FDMNs (Table 1). 

**Table 1:** Distribution of participants based on type of residency

Variables	Total *n* (%)	Type of residency		
		FDMNs *n* (= 58)	Host community *n* (= 596)	*p*-value
Age in years, median (IQR)	30.00 (25.00–41.00)	28.50 (23.25–35.75)	31.50 (25.00–43.00)	0.037*
Sex (male), n (%)	204 (31.19)	38 (65.52)	166 (27.85)	< 0.001
Marital status (single), n (%)	52 (7.95)	7 (12.07)	45 (7.55)	0.209**
Level of education (no formal education), n (%)	566 (86.54)	56 (96.55)	510 (85.57)	0.019
H/o blood transfusion (yes), n (%)	36 (5.50)	1 (1.72)	35 (5.87)	0.358**
H/o surgery (yes), n (%)	54 (8.28)	1 (1.72)	53 (8.92)	0.076**
H/o medicine intake for any illness (yes), n (%)	90 (13.82)	3 (5.26)	87 (14.65)	0.050
Family member suffering from HBV or HCV (yes), n (%)	6 (0.92)	0 (0.00)	6 (1.01)	> 0.999**
Vaccination against HBV (yes), n (%)	9 (1.38)	0 (0.00)	9 (1.51)	> 0.999**
Knowledge, (satisfactory), n (= 605) (%)	70 (11.57)	3 (5.17)	67 (12.25)	0.109
Attitude, (satisfactory), n (= 588) (%)	472 (80.27)	38 (71.70)	434 (81.12)	0.100
Practice, (satisfactory), n (= 654) (%)	320 (48.93)	22 (37.93)	298 (50.00)	0.079
HBV infection (positive), n (%)	7 (1.07)	1 (1.72)	6 (1.01)	0.480**
HCV infection (positive), n (%)	16 (2.45)	10 (17.24)	6 (1.01)	< 0.001**
HBV or HCV infection (positive), n (%)	23 (3.52)	11 (18.97)	12 (2.01)	< 0.001**

*HBV *Hepatitis B Viru, *HCV *Hepatitis C Virus

*Mann-Whitney U test

**Fisher’s exact test; FDMN: Forcibly Displaced Myanmar Nationals

We also found that age in years, sex, and level of education were significantly associated with the type of residency. No significant differences were observed between FDMNs and host community in the overall proportions of participants with satisfactory knowledge, attitude, or practice scores, although several individual KAP items differed significantly between the groups.

Simple binomial logistic regression examined associations between infection type and residency (Supplementary Fig. 1). HBV infection was not significantly associated with participant type (uOR: 0.58, p: 0.617, 95% CI: 0.07–4.90). In contrast, HCV infection was strongly associated with lower odds of infection among host community compared with FDMNs (uOR: 0.05, *p* < 0.001, 95% CI: 0.02–0.14). Overall HBV or HCV positivity was significantly higher among FDMNs than host community (uOR: 0.09, *p* < 0.001, 95% CI: 0.0-0.21).

Awareness of hepatitis B was generally higher than awareness of hepatitis C in both groups, although overall awareness remained low. None of the FDMNs were aware of the availability of the HBV vaccine, and only a small proportion recognised its preventive role compared with host community. For knowledge items common to both infections, awareness that HBV and HCV can affect individuals of any age was significantly lower among FDMNs than host community. Similarly, knowledge of mother-to-child transmission was lower among FDMNs (Table 2).

**Table 2:** Comparison of knowledge and practice level towards HBV or HCV infection between FDMNs and the host community

Items	Total *n* (%)	FDMN *n* (= 58) (%)	Host community *n* (= 596) (%)	*p*-value
Knowledge Questions				
* HBV-specific items*				
Have you ever heard of a disease termed hepatitis B? (yes)	117 (17.92)	6 (10.34)	111 (18.66)	0.115
Do you know what causes HBV infection? (yes)	23 (3.53)	2 (3.45)	21 (3.54)	> 0.999*
Is vaccination available for HBV? (yes)	58 (8.87)	0 (0.00)	58 (9.73)	0.006*
Is vaccination a method of preventing HBV? (yes)	96 (14.88)	1 (1.72)	95 (16.18)	0.003
* HCV-specific items*				
Have you ever heard of a disease termed hepatitis C? (yes)	68(10.41)	3 (5.17)	65 (10.92)	0.171
Do you know what causes HCV infection? (yes)	19 (2.91)	0 (0.00)	19 (3.20)	0.400*
* Common to both (HBV and HCV)*				
Can HBV and/or HCV infections result in chronic hepatitis and liver cancer? (yes)	171 (26.31)	9 (15.52)	162 (27.36)	0.051
Can HBV and/or HCV affect any age group? (yes)	191 (29.25)	10 (17.24)	181 (30.42)	0.035
Is HBV and/or HCV transmitted from mother to child? (yes)	119 (18.22)	4 (6.90)	115 (19.33)	0.019
Do you know if sexual intercourse is a mode of transmission? (yes)	115 (17.83)	5 (8.62)	110 (18.74)	0.055
Do you know if blood transfusion is a mode of transmission? (yes)	124 (19.28)	5 (8.62)	119 (20.34)	0.031
Do you know if infected needles are a mode of transmission? (yes)	88 (13.79)	5 (8.62)	83 (14.31)	0.231
Is avoiding unprotected sex a method of preventing HBV and/or HCV (yes)	110 (17.19)	5 (8.62)	105 (18.04)	0.070
Is material sterilization a method of preventing HBV and/or HCV? (yes)	69 (10.97)	3 (5.17)	66 (11.56)	0.138
Is proper disposal of sharp instruments a method of preventing HBV and/or HCV? (yes)	77 (12.2)	4 (6.90)	73 (12.74)	0.195
Is avoiding needle stick injury a method of preventing HBV and/or HCV? (yes)	92 (14.51)	5 (8.62)	87 (15.10)	0.181
Practice questions				
Have you done a screening for HBV and/or HCV? (yes)	28 (4.29)	0 (0.00)	28 (4.71)	0.163*
Are you aware of the appropriate intervals of the HBV (yes)	21 (3.29)	0 (0.00)	21 (3.6)	0.245*
Do you ask for screening before blood transfusion? (yes)	369 (57.39)	26 (44.83)	343 (58.63)	0.043
Do you ask for new syringe before use? (yes)	411 (64.12)	42 (72.41)	369 (63.29)	0.167
Do you ask your barber to change blade/or for safe equipment for ear or nose piercing? (yes)	539 (82.54)	47 (81.03)	492 (82.69)	0.751
Do you want to take precaution before dental procedure? (yes)	375 (58.41)	34 (59.65)	341 (58.29)	0.843
Have you ever participated in health education program related to HBV and/or HCV? (yes)	29 (4.46)	1 (1.75)	28 (4.72)	0.502*

Percentages are calculated based on the number of respondents to each item; denominators vary due to non-response

* Fisher’s exact test

We examined the preventive practices for HBV or HCV infection using 7 questions. Requesting blood screening before transfusion was reported more frequently among host community than FDMNs. Positive responses for all other practices, such as HBV or HCV screening, adherence to vaccine schedules, use of new syringes, use of safe equipment by barbers, precaution for dental procedures, and participation in health education, were comparable for both populations (Table 2).

We used 25 questions to evaluate attitudes toward HBV or HCV infection. A significantly higher percentage of the host community dealt with HBV or HCV-infected patients with caution, and more often feared spreading the infection to family members compared with FDMNs. After a diagnosis of infection, a higher proportion of FDMNs reported willingness to disclose their infection status to their parents and had a fear of isolation from society, compared with host community (Table 3).

**Table 3:** Attitudes towards HBV or HCV infection among FDMNs and host community

Attitude items	Total *n* (%)	FDMNs (*n* = 58) (%)	Host community (*n* = 596) (%)	*p*-value
If you have ever been diagnosed with HBV or HCV virus infection, would you be willing to be treated? (yes)	625 (95.71)	55 (94.83)	570 (95.80)	0.728
Would you share a meal with an HCV or HBV-infected person? (yes)	89 (13.67)	7 (12.28)	82 (13.80)	0.749
Should patients with HBV and/or HCV be allowed to work routinely? (yes)	62 (9.60)	6 (10.34)	56 (9.52)	0.840
Is vaccination against HBV necessary? (yes)	610 (94.28)	54 (93.10)	556 (94.40)	0.564*
How do you deal with HBV and/or HCV patient?				
I avoid dealing with them (yes)**	198 (30.46)	34 (58.62)	164 (27.70)	< 0.001
I deal with them cautiously (yes)**	526 (81.42)	30 (52.63)	496 (84.21)	< 0.001
I deal with them normally (yes)	48 (7.40)	2 (3.51)	46 (7.77)	0.422*
What would be your reaction if you were diagnosed with HBV and/or HCV?				
Fear (yes)	332 (51.08)	32 (55.17)	300 (50.68)	0.513
Shame (yes)**	121 (18.62)	8 (13.79)	113 (19.09)	0.323
Sadness (yes)**	323 (49.69)	26 (44.83)	297 (50.17)	0.437
What will you do if you have symptoms of HBV and/or HCV?				
Self-treatment (yes)**	617 (5.08)	0 (0.00)	559 (5.57)	0.065
Going to a health facility (yes)	613 (94.02)	57 (98.28)	556 (93.60)	0.152
Going to homoeopathic healer (yes)**	12 (1.83)	0 (0.00)	12 (2.01)	0.613*
Going to a traditional healer (yes)**	17 (2.61)	1 (1.72)	16 (2.70)	> 0.999*
What will worry you most if you were diagnosed with HBV and/or HCV?				
Fear of death (yes)	311 (47.99)	22 (39.29)	289 (48.82)	0.172
Fear that the disease may spread to a family member (yes)	472 (72.84)	27 (48.21)	445 (75.17)	< 0.001
The treatment cost (yes)	187 (28.77)	19 (33.93)	168 (28.28)	0.372
Isolation from society (yes)**	75 (11.56)	11 (19.64)	64 (10.79)	0.048
Whom would you inform about your illness if your test were positive?				
Physician (yes)	599 (92.01)	51 (87.93)	548 (92.41)	0.230
Spouse (yes)	335 (51.30)	23 (39.66)	312 (52.44)	0.063
Parents (yes)	63 (9.65)	15 (25.86)	48 (8.07)	< 0.001
Child (yes)	38 (5.81)	3 (5.17)	35 (5.87)	> 0.999*
Other relatives (yes)	9 (1.38)	1 (1.72)	8 (1.34)	0.569*
Friends (yes)	3 (0.46)	0 (0.00)	3 (0.50)	> 0.999*
No one (yes)**	603 (0.99)	0 (0.00)	548 (1.08)	0.438

Percentages are calculated based on the number of respondents to each item; denominators vary due to non-response

*p-value from Fisher’s exact test reported

**indicates negative attitude

Supplementary Table 1 presents a comparison of their safe sex behaviour and HBV or HCV infection rates and showing a significantly lower percentage of the FDMNs knew or used any barrier method for sexual intercourse. 

## Discussion

To our knowledge, this study is the first to compare HBV and HCV infection prevalence between FDMNs and host community while concurrently assessing knowledge, attitudes, and practices regarding these infections. A key finding of this study was that a significantly higher percentage of FDMNs were affected by HBV or HCV infection. The comparison of satisfactory knowledge, attitude, and practice between host community and FDMNs indicates that host community exhibited a higher proportion of satisfactory levels across all domains, although none of the observed differences reach statistical significance.

The FDMNs experienced prolonged economic and political disadvantage, with limited access to health education [17]. The high prevalence of HCV infection in this population may increase the risk of progressive liver dysfunction and cirrhosis. Among host community, HCV prevalence among the host community was approximately 1%, substantially lower than that observed among FDMNs [3]. Several factors may be attributed to this higher prevalence of HCV relative to HBV among the FDMNs, which may include prior levels of exposure as well as unsafe medical practices. A large study, conducted among 2000 participants in 2017, reported that 213 (11.0%) tested positive for anti-HCV and 82 (4.0%) emerged positive for HBsAg. The study also reported that 0.4% tested positive for both HBV and HCV [2]. Among adult females, anti-HCV was found to be positive in 26% cases. The study also reported a higher prevalence of HbsAG and anti-HCV positive cases among the pregnant women, highlighting the potential risk of vertical transmission [2]. Variations in prevalence estimates across studies may reflect differences in study design, sampling approaches, study settings, participant characteristics, sample sizes, and diagnostic approaches, including the distinction between seropositivity and active infection.

The findings indicate generally low KAP regarding HBV and HCV in both populations, particularly among FDMNs, among whom awareness was comparatively lower and fewer than one-fifth of participants reported having heard of HBV or HCV. Knowledge regarding causes, transmission routes, and prevention remained limited. Awareness of HBV vaccination was particularly low among FDMNs, while awareness of HCV was generally lower than that of HBV in both populations. A recent systematic review reported four main barriers to vaccination. These four barriers included cultural beliefs, limited access to healthcare, lack of knowledge, and vaccine hesitancy, primarily due to safety concerns [18]. Additionally, lower awareness of HCV compared to HBV may reflect differences in public health messaging, particularly the emphasis on HBV vaccination.

Knowledge of transmission routes, including sexual contact and blood transfusion, remained limited. Awareness of preventive practices remained low, with FDMNs consistently less informed. Practice-related indicators reflected this knowledge gap. Only 4.3% of participants had ever been screened for HBV or HCV, with none from the FDMNs reporting screening. Some preventive practices were reported more frequently among FDMNs, although differences were not statistically significant [19].

The current study observed lower adherence to precautionary measures among FDMNs when interacting with infected individuals, and a lower proportion expressed concern regarding the transmission of infection to household members. However, FDMNs more frequently reported willingness to disclose infection status to their parents, indicating a distinct pattern of intra-family communication that warrants further investigation. The consistently low uptake of viral screening across the study population, particularly among FDMNs, underscores the urgent need for targeted interventions focusing on sustained awareness campaigns and expanded access to screening services for this marginalised community.

The higher prevalence of HBV and HCV among FDMNs may reflect cumulative past exposures and structural vulnerabilities. These differences may not be fully explained by current KAP scores alone [20]. Limited access to healthcare, vaccination, screening, and health information may have increased infection risk among FDMNs. Crowded living conditions within refugee settings may also have contributed. Furthermore, current KAP measures may not fully capture all behavioural, environmental, and healthcare-related determinants of transmission.

The study had several limitations. First, there were important sampling and generalisability limitations. The relatively small number of FDMNs, substantial imbalance in group sizes, and hospital-based convenience sampling approach may have limited statistical power, reduced the precision of comparisons, and introduced selection bias. In addition, sex distribution differed considerably between the groups, and residual confounding cannot be excluded. Second, diagnostic and data-related limitations should be acknowledged. Rapid immunoassays were used without confirmatory ELISA or PCR testing, which may have resulted in misclassification, and anti-HCV seropositivity may not necessarily indicate active infection. The absence of detailed socioeconomic variables limited assessment of their influence on KAP and infection outcomes. Finally, longitudinal follow-up of participants with HBV or HCV was not possible.

## Conclusion

FDMNs demonstrated a substantially higher burden of HBV and HCV compared with the host community, while KAP regarding viral hepatitis remained low in both populations. These findings highlight the need for targeted health education and culturally appropriate awareness programmes, particularly within refugee settings. Strengthening access to screening, diagnostic services, and linkage to care may help improve early detection and reduce future transmission. Integration of refugee health services within national hepatitis elimination strategies will be important to support equitable prevention and care for both refugee and host populations.

## Supplementary Information

Supplementary Material 1: Supplementary Table 1. Comparison of safe sex behaviours between FDMNs and the host community. Percentages are calculated based on the number of respondents to each item; denominators vary due to non-response. *:Fisher’s exact test. Supplemental Figure 1. Simple binomial logistic regression showing the association between infection types and type of residency.

## Data Availability

The datasets generated and/or analysed during the current study are available from the corresponding author on reasonable request. All data are de-identified to ensure participant confidentiality. Data related to this manuscript are available upon request, and researchers who meet the criteria for access to confidential data may contact Ms. Shiblee Sayeed (shiblee_s@icddrb.org) of the Research Administration of icddr, b (http://www.icddrb.org/).
