# Supplementary material for: The hidden burden of viral hepatitis: a comparative study of KAP and infections among rohingya refugees and host communities in Bangladesh
Source: BMC Public Health. 2026 May 25;26:2174. doi: 10.1186/s12889-026-27880-6 (PMC13383131; doi:10.1186/s12889-026-27880-6)
Supplement: Supplementary file 1 — Supplementary Material 1: Supplementary Table 1. Comparison of safe sex behaviours between FDMNs and the host community. Percentages are calculated based on the number of respondents to each item; denominators vary due to non-response. *:Fisher’s exact test. Supplemental Figure 1. Simple binomial logistic regression showing the association between infection types and type of residency. [file 12889_2026_27880_MOESM1_ESM.docx]

**Supplementary Table 1. Comparison of safe sex behaviours between FDMNs and the host community**

|  | **Total**  **n (%)** | **FDMNs**  **(n=58) (%)** | **Host community**  **(n=596) (%)** | **p-value** |
| --- | --- | --- | --- | --- |
| Do you use or know about any family planning methods? (yes) | 575 (87.92) | 51 (87.93) | 524 (87.92) | 0.998 |
| Do you use or know of any barrier method like condoms during sexual intercourse (yes) | 179 (27.41) | 6 (10.53) | 173 (29.03) | 0.003 |
| History of any unprotected sexual intercourse? (yes) | 11 (1.68) | 0 (0.00) | 11 (1.85) | 0.611* |

*Percentages are calculated based on the number of respondents to each item; denominators vary due to non-response.*

**:Fisher’s exact test*


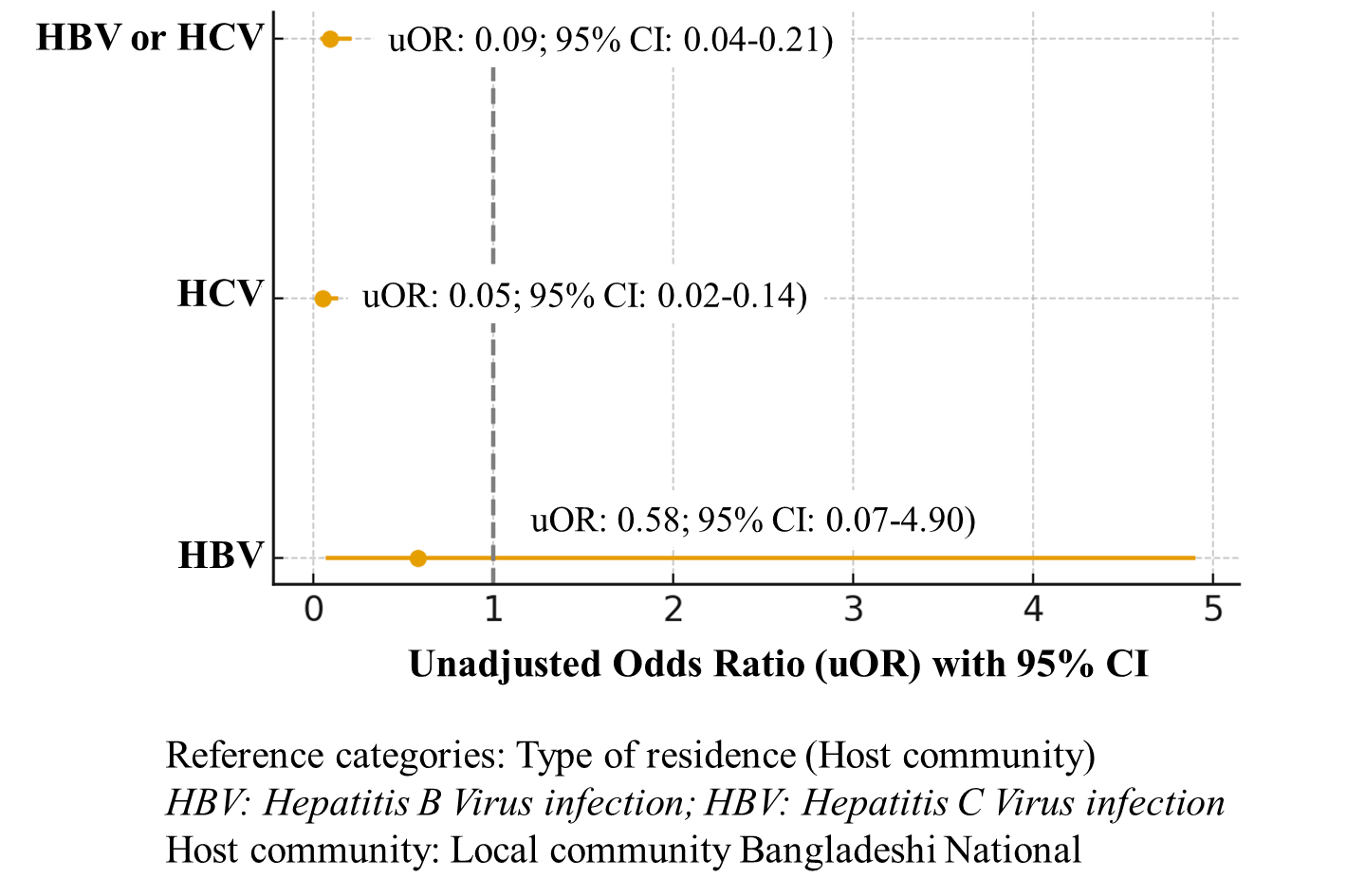


**Supplemental Figure 1.** Simple binomial logistic regression showing the association between infection types and type of residency
